# Association between severity of nonalcoholic fatty liver disease and major adverse cardiovascular events in patients assessed by coronary computed tomography angiography

**DOI:** 10.1186/s12872-024-03880-5

**Published:** 2024-05-21

**Authors:** Rongchao Shi, Xuemei Li, Kui Sun, Fangyuan Liu, Bing Kang, Yilin Wang, Ying Wang, Baosen Zhu, Xinya Zhao, Zhiqiang Liu, Ximing Wang

**Affiliations:** 1 Department of Radiology, Shandong Provincial Hospital, Shandong University, Jinan, Shandong Province China; 2 Department of Gastroenterology, Shandong Provincial Hospital, Shandong University, Jinan, Shandong Province China; 3 Department of Gastroenterology, Heze Municipal Hospital, Heze, Shandong Province China; 4 Department of Radiology, Shandong Provincial Hospital Affiliated to Shandong First Medical University, Jinan, Shandong Province China

**Keywords:** Nonalcoholic fatty liver disease, Severity, Computed tomography, Coronary computed tomography angiography, Major adverse cardiovascular events

## Abstract

**Background:**

The effect of nonalcoholic fatty liver disease (NAFLD) on major adverse cardiovascular events (MACEs) can be influenced by the degree of coronary artery stenosis. However, the association between the severity of NAFLD and MACEs in patients who underwent coronary computed tomography angiography (CCTA) is unclear.

**Methods:**

A total of 341 NAFLD patients who underwent CCTA were enrolled. The severity of NAFLD was divided into mild NAFLD and moderate-severe NAFLD by abdominal CT results. The degree of coronary artery stenosis was evaluated by using Coronary Artery Disease Reporting and Data System (CAD-RADS) category. Cox regression analysis and Kaplan–Meier analysis were used to assess poor prognosis.

**Results:**

During the follow-up period, 45 of 341 NAFLD patients (13.20%) who underwent CCTA occurred MACEs. The severity of NAFLD (hazard ratio [HR] = 2.95[1.54–5.66]; *p* = 0.001) and CAD-RADS categories 3–5 (HR = 16.31[6.34–41.92]; *p* < 0.001) were independent risk factors for MACEs. The Kaplan–Meier analysis showed that moderate to severe NAFLD patients had a worsen prognosis than mild NAFLD patients (log-rank *p* < 0.001). Moreover, the combined receiver operating characteristic curve of the severity of NAFLD and CAD-RADS category showed a good predicting performance for the risk of MACEs, with an area under the curve of 0.849 (95% CI = 0.786–0.911).

**Conclusion:**

The severity of NAFLD was independent risk factor for MACEs in patients with obstructive CAD, having CAD-RADS 3–5 categories on CCTA.

**Supplementary Information:**

The online version contains supplementary material available at 10.1186/s12872-024-03880-5.

## Introduction

Coronary computed tomography angiography (CCTA) has become a first-line recommendation for screening patients with suspected coronary artery disease (CAD) and is widely used to detect coronary stenosis and analysis plaque characteristics. Due to CCTA can monitor early changes of CAD, it has become a new and valuable prognostic tool for evaluating major adverse cardiovascular events (MACEs) [1, 2]. Previous studies showed the prognosis value of CCTA in nonalcoholic fatty liver disease (NAFLD) patients [3, 4] and NAFLD patients with CAD have a high risk of MACEs. Because of the prognosis of CAD patients depending on the degree of coronary artery stenosis, the effect of NAFLD on MACEs may be influenced by the degree of coronary artery stenosis [3].

The severity of NAFLD is independently associated with carotid atherosclerosis [5, 6] and moderate to severe NAFLD patients had a higher incidence of future ischemic stroke events [7]. The severity of NAFLD also closely correlates with cardiac complications, such as aortic valve calcification, arrhythmias, epicardial fat thickness, and left ventricular hypertrophy et al. [8–10]. And moderate to severe NAFLD patients with hypertension have a higher risk of all-cause death compared with mild NAFLD patients [11, 12]. However, the association is unclear between the severity of NAFLD and MACEs in patients with different degrees of coronary artery stenosis.

Therefore, our current study was to investigate the association between the severity of NAFLD and MACEs in patients assessed by CCTA and evaluated predicting performance of the severity of NAFLD and degree of coronary artery stenosis for the risk of MACEs.

## Methods

### Patients

**Fig. 1:**
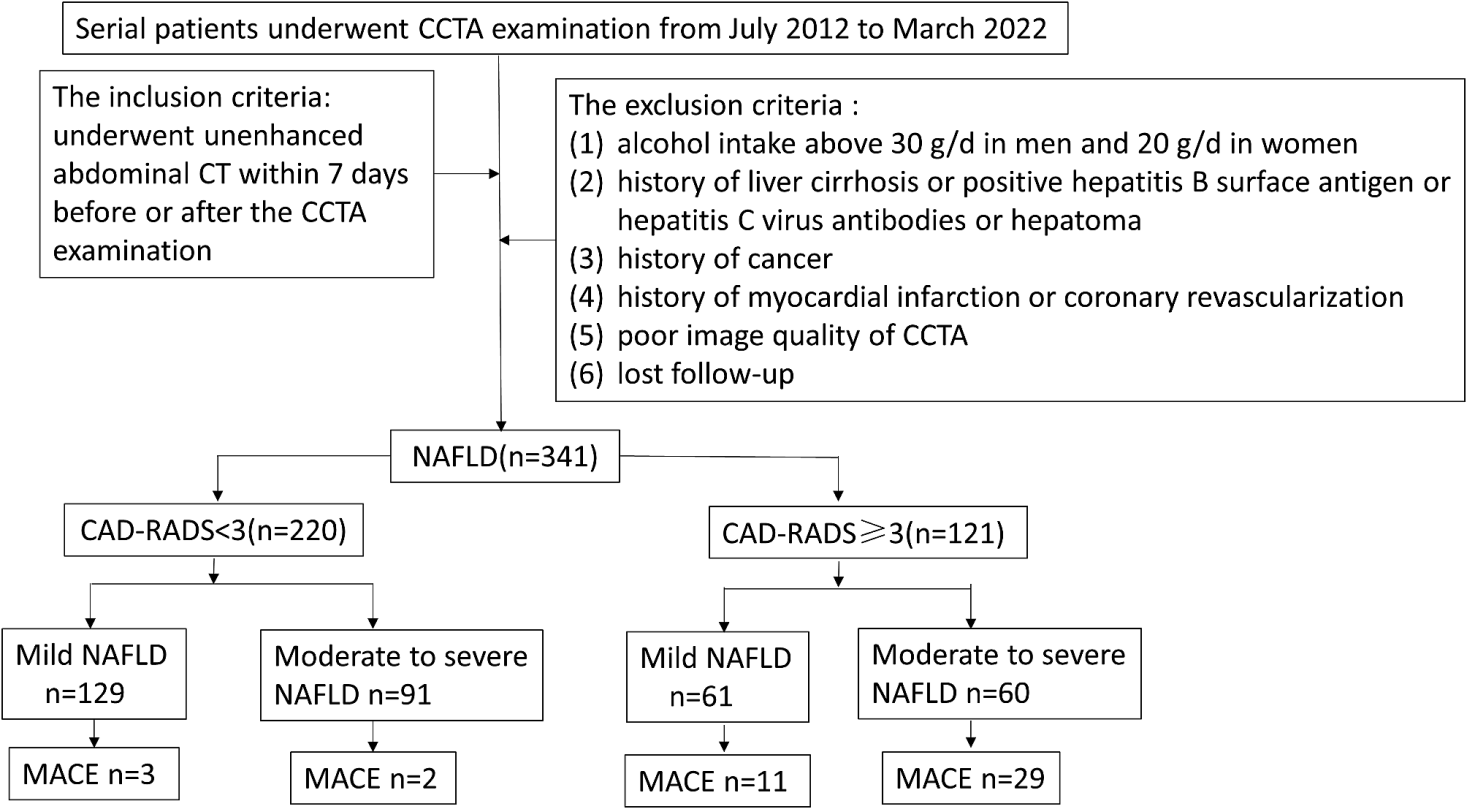
The flowchart of inclusion and exclusion criteria. CCTA, coronary CT angiography; NAFLD, non-alcoholic fatty liver disease; CAD-RADS, coronary artery disease reporting and data system; MACEs, major adverse cardiovascular events

We retrospectively analyzed the continuous patients who received CCTA examination and simultaneously underwent unenhanced abdominal CT within one week in our institution between July 2012 and March 2022. The exclusion criteria were as follows: [1] a history of alcohol abuse, with outweigh 30 g/d in men and 20 g/d in women [13]; [2] history of liver cirrhosis or positive hepatitis B surface antigen or hepatitis C virus antibodies or hepatoma; [3] history of cancer; [4] previous history of myocardial infarction or coronary artery revascularization; [5] poor image quality of CCTA; and [6] individuals who lost follow-up. The flowchart of inclusion and exclusion criteria for this study is shown in Fig. 1. Finally, a total of 341 NAFLD patients were included in this research. The non-NAFLD patients incorporated 145 patients who also underwent CCTA examination and unenhanced abdominal CT as a control group.

The requirement for informed consent was waived by Ethics Committee of Shandong Provincial Hospital Affiliated to Shandong First Medical University.

### CCTA image acquisition

Routine coronary computed tomography angiography (CCTA) dates were obtained by dual-source CT scanner (Aquilion ONE, TOSHIBA; Somatom Flash or Force, Siemens Healthineers). Before scanning, all patients sprayed nitroglycerin sublingually to dilate the coronary artery and received breath-holding training to reduce respiratory motion artifacts. CCTA was performed by using a bolus tracking technique, with regions of interest placed in the root of the aorta. Detailed CCTA parameters are listed in Supplementary E1.

### Image reconstruction and assessment of CCTA

The degree of coronary artery stenosis was evaluated by using the Coronary Artery Disease Reporting and Data System (CAD-RADS) category [14]. A dedicated plaque analysis software (Coronary Plaque Analysis, version 5.0.0, Siemens Healthineers, Germany) was used to measure the quantitative plaque parameters. The quantitative coronary perivascular fat attenuation index (FAI) was evaluated by using a dedicated FAI analysis software (Easy FAI, version 1.2, ShuKun, China).The detailed image evaluation were shown Supplementary E2.

Two radiologists with 20 years and 10 years of cardiac imaging experience, who were blinded to the NAFLD status and clinical outcome, independently analyzed imaging. The measured values were used for further analysis. Stenosis of the left main trunk ≥ 50% or non-left main trunk ≥ 70% is severe coronary artery stenosis, for which revascularization is often required. Coronary artery stenosis with 40–69% are moderately narrow and usually require further functional evaluation [15].

### The severity of NAFLD definition and measurement

The severity of NAFLD was defined by liver attenuation minus spleen attenuation (L-S) on unenhanced CT. We defined L-S≤-10HU to indicate moderate to severe NAFLD [16]. The reviewers who measured liver and spleen attenuation were blinded to the patient’s clinical information. Liver and spleen Hounsfield units were obtained by drawing 3 circular regions of interest (ROI) with an area of at least 200 mm^2^ on different axial levels [3], and the ROI values were calculated as the average of the three measurements. We selected carefully sample homogeneous areas representative of the parenchyma, avoiding blood vessels, bile ducts, calcification, cyst, focal lesions, and surface margins.

### Clinical outcomes

Clinical follow-up times were started after the time of the CCTA examination. Follow-up clinical dates were acquired by reviewing medical records or telephone interviews. The primary endpoint was the occurrence of MACEs, which were defined as all-cause death, nonfatal myocardial infarction, heart failure, and revascularization. Revascularization included percutaneous coronary intervention and coronary artery bypass grafting.

### Statistical analysis

The intraclass correlation coefficient (ICC) was used to determine the interobserver reproducibility of the liver, spleen attenuation measurement, and lesion length. The agreement of CAD-RADS categories was tested with the Cohen k value. The SPSS version 26.0 and R version 4.0.1 software were used for statistical analysis. Continuous dates were depicted as mean ± standard deviation (SD) or median and quartiles, while categorical variables were showed as frequency with percentages.

Cox regression analysis was applied to reveal association between the severity of NAFLD and MACEs. The Kaplan-Meier analysis was used to assess the prognosis of NAFLD patients. Receiver operating characteristic (ROC) curves and area under the curve (AUC) were performed to assess the predictive performance of the severity of NAFLD and CAD-RADS category for MACEs in patients assessed by CCTA. All statistical tests were two-sided, with *p* < 0.05 considered statistically significant.

## Results

### Clinical baseline characteristics of patients

In this study, the median age of the registered patients was 59 years and 138 (40.47%) of patients were men. The demographic characteristics and laboratory data of the non-NAFLD patients, mild NAFLD patients, and moderate to severe NAFLD patients were shown in Table 1. Compared with mild NAFLD patients, the moderate to severe NAFLD patients had higher aspartate aminotransferase (AST), alanine aminotransferase (ALT), total cholesterol (TC), and low-density lipoprotein cholesterol (LDL-C) (all *p* < 0.05). Both groups had no statistical difference in the presence of diabetes mellitus, and level of gamma glutamyl transferase (GGT), triglyceride (TG), high-density lipoprotein cholesterol (HDL-C), glucose (GLU), and uric acid (URIC) (all *p* > 0.05). The NAFLD patients were more likely to have diabetes mellitus and had higher AST, ALT, GGT, TG, LDL-C, GLU, URIC, and lower HDL-C than that of non-NAFLD patients (all *p* < 0.05).

**Table 1:** Baseline clinical data of the study population

Characteristic		non-NAFLD (*n* = 145)	Mild NAFLD (*n* = 190)	Moderate to severe NAFLD (*n* = 151)	All patients (*n* = 341)
Age(y)		62(56–68)	59.5(52.5–66.5)†	59(51–67)†	59(51.5–66.5)
Men(%)		46(31.72)	75(39.47)	63(41.72)	138(40.47)
**Risk of factor**					
History of smoking		20(13.79)	25(13.16)	26(17.22)	51(14.96)
Hypertension		85(58.62)	130(68.42)	95(62.91)	225(65.98)
Diabetes mellitus		30(20.69)	71(37.37)†	61(40.40)†	132(38.71)†
History of coronary heart disease		34(23.45)	62(32.63)	37(24.50)	99(29.03)
Medication history		44(30.34)	50(26.32)	39(25.83)	89(26.10)
Family history of coronary heart disease		15(10.34)	23(12.11)	13(8.61)	36(10.56)
**Laboratory tests**					
AST (U/L)		19(16.5–21.5)	22.5(17.0–28.0)†	26.0(19.0–33.0)†*	24.0(18.0–30.0)†
ALT (U/L)		15(11.0–19.0)	25.0(16.5–33.5)†	31.0(19.5–42.5)†*	27.0(17.5–36.5)†
GGT (U/L)		20(13.8–26.3)	29.5(18.5–40.5)†	32.0(22.5–41.5)†	31.0(20.5–41.5)†
TG (mmol/L)		1.27(0.91–1.63)	1.8(1.15–2.45)†	2.12(1.31–2.94)†	1.92(1.19–2.66)†
TC (mmol/L)		4.75(4.03–5.48)	4.74(3.89–5.59)	5.05(4.29–5.81)*	4.86(4.04–5.69)
HDL-C (mmol/L)		1.29(1.08–1.50)	1.13(0.93–1.34)†	1.11(0.93–1.29)†	1.11(0.92–1.31)†
LDL-C (mmol/L)		2.77(2.23–3.31)	2.89(2.30–3.49)	3.15 (2.54–3.76)†*	3.02(2.40–3.64)†
GLU (mmol/L)		5.52(4.95–6.10)	6.12(5.06–7.19)†	6.49(5.10–7.88)†	6.24(5.00-7.48)†
BUN(mmol/L)		4.80(3.40–5.65)	5.00(4.09–5.92)	5.10(4.20-6.00)†	5.00(4.05–5.95)
CREA(µmol/L)		60.24(53.09–67.39)	60.90(49.72–72.09)	59.50(48.50–70.50)	60.10(49.53–70.68)
URIC(µmol/L)		289(239.5-338.5)	338.5(279–398)†	344(278.5-409.5)†	340(279–401)†
**CAD-RADS category**			0.001†		0.003†
0		61(42.07)	43(22.63)	41(27.15)	84(24.63)
1		18(12.42)	43(22.63)	22(14.57)	65(19.06)
2		18(12.42)	43(22.63)	28(18.54)	71(20.82)
3		17(11.72)	28(14.74)	23(15.23)	51(14.96)
4 A		28(19.31)	30(15.79)	32(21.19)	62(18.18)
4B		1(0.69)	2(1.05)	3(1.99)	5(1.47)
5		2(1.38)	1(0.53)	2(1.32)	3(0.88)
FAI/HU	LAD	-83.57 ± 9.38	-82.73 ± 8.70	-80.05 ± 9.67*	-81.54 ± 9.22†
	LCX	-82.40 ± 10.23	-79.24 ± 9.01	-77.23 ± 7.88*	-78.35 ± 8.57†
	RCA	-85.26 ± 11.65	-83.43 ± 9.63	-81.30 ± 10.13*	-82.49 ± 9.90†

*Note* Data are presented as median (interquartile range) or number (percentage). †*p* < 0.05 (compared with non-NAFLD). *p < 0.05 (compared with mild NAFLD)

*Abbreviations* NAFLD, nonalcoholic fatty liver disease; ALT, alanine aminotransferase; AST, aspartate aminotransferase; GGT, gamma glutamyl transferase; TG, triglyceride; TC, total cholesterol; HDL-C, high-density lipoprotein cholesterol; LDL-C, low-density lipoprotein cholesterol; GLU, glucose; BUN, blood urea nitrogen; CREA, creatinine; URIC, uric acid; CAD-RADS, Coronary Artery Disease Reporting and Data System; FAI, fat attenuation index; LAD, left anterior descending artery; LCX, left circumflex artery; RCA, right coronary artery

### Quantitative parameters (plaque features and FAI) based on CCTA were compared among the non-NAFLD, mild NAFLD, and moderate to severe NAFLD patients

Though no statistically significant difference in CAD-RADS category was observed between the mild NAFLD patients and moderate to severe NAFLD patients, the latter had a higher proportion of CAD-RADS categories 3–5 than the former. FAI of three coronary arteries had statistical difference between mild NAFLD patients and moderate to severe NAFLD patients (all *p* < 0.05). The detailed results were shown in Table 1. As presented in Table 2, moderate to severe NAFLD patients had lower calcified plaque volume and calcified plaque volume ratio compared with mild NAFLD patients (all *p* < 0.05). NAFLD patients had longer lesions range and higher fibrotic plaque volume and fibrotic plaque volume ratio than non-NAFLD patients. FAI of three coronary arteries also had statistical difference between NAFLD patients and non-NAFLD patients (all *p* < 0.05) (Table 1).

**Table 2:** The quantitative parameters of plaques

	non-NAFLD(*n* = 84)	Mild NAFLD(*n* = 147)	Moderate to severe NAFLD(*n* = 110)	All Patients(*n* = 257)
lesion length/mm	12.85(9.37–16.34)	18.00(13.40–22.60)†	16.50(11.93–21.08)†	17.30(12.60–22.00)†
TPV/ mm^3^	108.40(43.17-173.62)	124.64(64.56-184.73)	124.78 (65.02-184.54)	124.64(65.70-183.59)
CPV/ mm^3^	39.08(8.28–69.89)	33.30(12.85–53.75)	18.59(0-39.93)†*	28.47(7.10-49.84)†
CPR/%	38.35(21.84–54.87)	24.70(11.90–37.50)	21.15(9.52–32.79)†*	23.00(10.70–35.30)†
LPV/ mm^3^	2.89(0-6.77)	1.34(0-4.51)	3.03(0-7.05)	2.05(0-5.31)
LPR/%	2.10(0-5.33)	1.20(0–3.00)	2.15(0-4.85)	1.50(0-3.75)
FPV/ mm^3^	58.22(23.94–92.51)	83.47(37.29-129.65)†	86.30(47.88-124.73)†	84.24(43.12-125.37)†
FPR/%	55.25(40.44–70.07)	70.80(59.15–82.45)†	75.50(64.99–86.02)†	73.20(62.04–84.36)†

*Note*- Data are showed as means ± standard deviations or median (interquartile range)

†*p* < 0.05 (compared with non-NAFLD). *p < 0.05 (compared with mild NAFLD)

*Abbreviations* TPV, total plaque volume; CPV, calcified plaque volume; CPR, calcified plaque volumes ratio; LPV, lipid plaque volume; LPR, lipid plaque volume ratio; FPV, fibrotic plaque volume; FPR, fibrotic plaque volume ratio

The interobserver reliabilities of the CAD-RADS category at CCTA was great (k = 0.96). The interobserver reliabilities of the liver and spleen CT attenuation measurement, and lesion length were excellent (ICC = 0.98 [95% confidence interval CI = 0.97–0.99], 0.93 [95% CI = 0.91–0.94] and 0.98 [95% CI = 0.96–0.99], respectively).

### The severity of NAFLD and CAD-RADS categories 3–5 were significantly independent risk factors for MACEs

The median time of follow-up was 28 months (interquartile range, 10.0–46.0 months). During the follow-up period, 45 of 341 patients (13.20%) had occurred MACEs in patients assessed by CCTA. On univariate analysis, men (hazard ratio [HR] = 1.99; 95% CI = 1.10–3.59; *p* = 0.022), history of smoking (HR = 3.23; 95% CI = 1.74–6.01; *p* < 0.001), CREA (HR = 1.02; 95% CI = 1.00–1.03; *p* = 0.014), severity of NAFLD (HR = 3.32; 95% CI = 1.75–6.30; *p* < 0.001), and CAD-RADS categories 3–5 (HR = 18.79; 95% CI = 7.39–47.75; *p* < 0.001) were significantly associated with MACEs. On multivariate analysis, the severity of NAFLD (HR = 2.95; 95% CI = 1.54–5.66; *p* = 0.001) and CAD-RADS categories 3–5 (HR = 16.31; 95% CI = 6.34–41.92; *p* < 0.001) were independent risk factors for MACEs. Detailed results were provided in Table 3.

**Table 3:** Cox regression analysis of variables associated with the risk of MACEs in patients assessed by CCTA

Variables		Univariate		Multivariate	
		HR (95%CI)	P Value	HR (95%CI)	P Value
Age(y)		1.01(0.99–1.04)	0.335	NA	
Men		1.99(1.10–3.59)	0.022	0.95(0.46–1.96)	0.889
**Risk of factor**					
History of smoking		3.23(1.74–6.01)	< 0.001	1.77 (0.89–3.52)	0.102
Hypertension		0.87 (0.47–1.60)	0.651	NA	
Diabetes mellitus		1.34(0.74–2.40)	0.336	NA	
History of coronary heart disease		0.84(0.43–1.63)	0.609	NA	
Medication history		1.28(0.68–2.42)	0.448	NA	
Family history		0.62(0.19–2.01)	0.425	NA	
**Laboratory tests**					
AST (U/L)		1.00(0.98–1.02)	0.692	NA	
ALT (U/L)		1.00(0.99–1.01)	0.851	NA	
GGT (U/L)		1.00(0.99–1.01)	0.585	NA	
TG (mmol/L)		1.02(0.86–1.22)	0.804	NA	
TC (mmol/L)		0.95(0.76–1.20)	0.675	NA	
HDL-C (mmol/L)		0.46(0.14–1.48)	0.193	NA	
LDL-C (mmol/L)		1.01(0.74–1.38)	0.938	NA	
GLU (mmol/L)		1.08(0.98–1.20)	0.120	NA	
BUN(mmol/L)		1.00(0.95–1.05)	0.944	NA	
CREA(µmol/L)		1.02(1.00-1.03)	0.014	1.02(1.00-1.04)	0.096
URIC(µmol/L)		1.00(1.00–1.00)	0.117	NA	
**CAD-RADS category**					
0–2		Reference			
3–5		18.79(7.39–47.75)	< 0.001	16.31(6.34–41.92)	< 0.001
**Severity of NAFLD**		3.32(1.75–6.30)	< 0.001	2.95(1.54–5.66)	0.001
**Plaque parameters**					
lesion length/mm		1.03(0.97–1.08)	0.344	NA	
TPV/ mm^3^		1.00(1.00–1.00)	0.072	NA	
CPV/ mm^3^		1.01(1.00-1.01)	0.400	NA	
CPR/%		1.00(0.98–1.02)	0.975	NA	
LPV/ mm^3^		1.02(0.99–1.06)	0.190	NA	
LPR/%		1.03(0.98–1.09)	0.223	NA	
FPV/ mm^3^		1.00(1.00-1.01)	0.199	NA	
FRP/%		1.00(0.98–1.02)	0.779	NA	
FAI/HU	LAD	1.02(0.98–1.05)	0.363	NA	
	LCX	1.03(1.00-1.05)	0.096	NA	
	RCA	1.03(1.00-1.06)	0.069	NA	

*Abbreviations* HR, hazard ratio; CI, confidence interval; NAFLD, nonalcoholic fatty liver disease; MACEs, major adverse cardiovascular events; CCTA, coronary computed tomographic angiography; CAD-RADS, Coronary Artery Disease Reporting and Data System; ALT, alanine aminotransferase; AST, aspartate aminotransferase; GGT, gamma glutamyl transferase; TG, triglyceride; TC, total cholesterol; HDL-C, high-density lipoprotein cholesterol; LDL-C, low-density lipoprotein cholesterol; GLU, glucose; BUN, blood urea nitrogen; CREA, creatinine; URIC, uric acid; FAI, fat attenuation index; TPV, total plaque volume; CPV, calcified plaque volume; CPR, calcified plaque volumes ratio; LPV, lipid plaque volume; LPR, lipid plaque volume ratio; FPV, fibrotic plaque volume; FPR, fibrotic plaque volume ratio; FAI, fat attenuation index; LAD, left anterior descending artery; LCX, left circumflex artery; RCA, right coronary artery

**Fig. 2:**
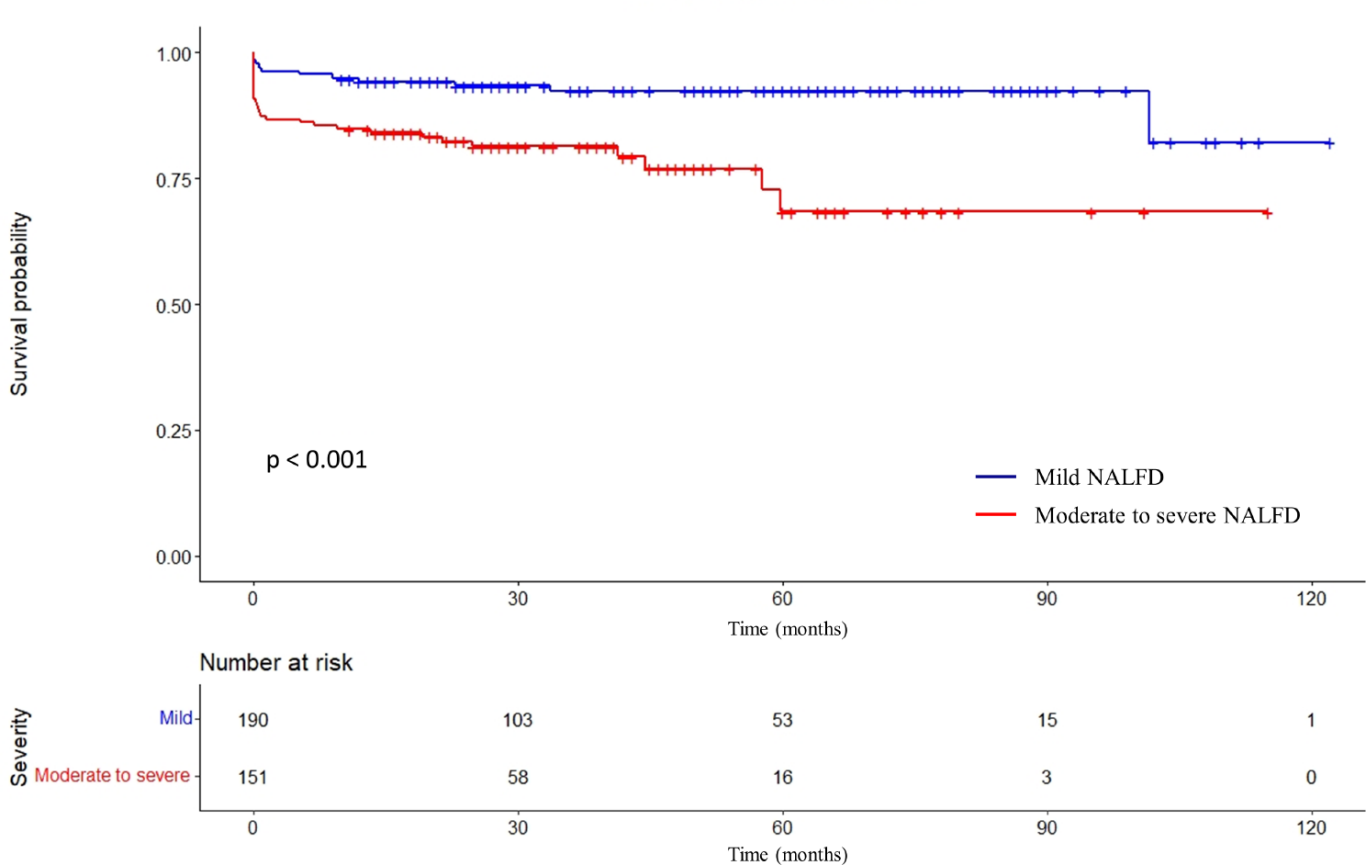
Kaplan–Meier curve of different severity of NAFLD in patients assessed by CCTA. Survival rate of moderate to severe NAFLD patients compared with mild NAFLD patients

The Kaplan–Meier curves analysis was used to compare the survival rate of the two groups and further evaluate their poor prognosis (Fig. 2). The 1-, 3-, and 5-year survival rates in the moderate to severe NAFLD patients were significantly lower than mild NAFLD patients (84.8%, 81.4%, and 68.4% vs. 94.2%, 92.5%, and 92.5%, respectively). The moderate to severe NAFLD patients had a worsen prognosis than mild NAFLD patients (log-rank *p* < 0.001).

### Performance of the severity of NAFLD and CAD-RADS category for predicting the risk of MACEs

**Fig. 3:**
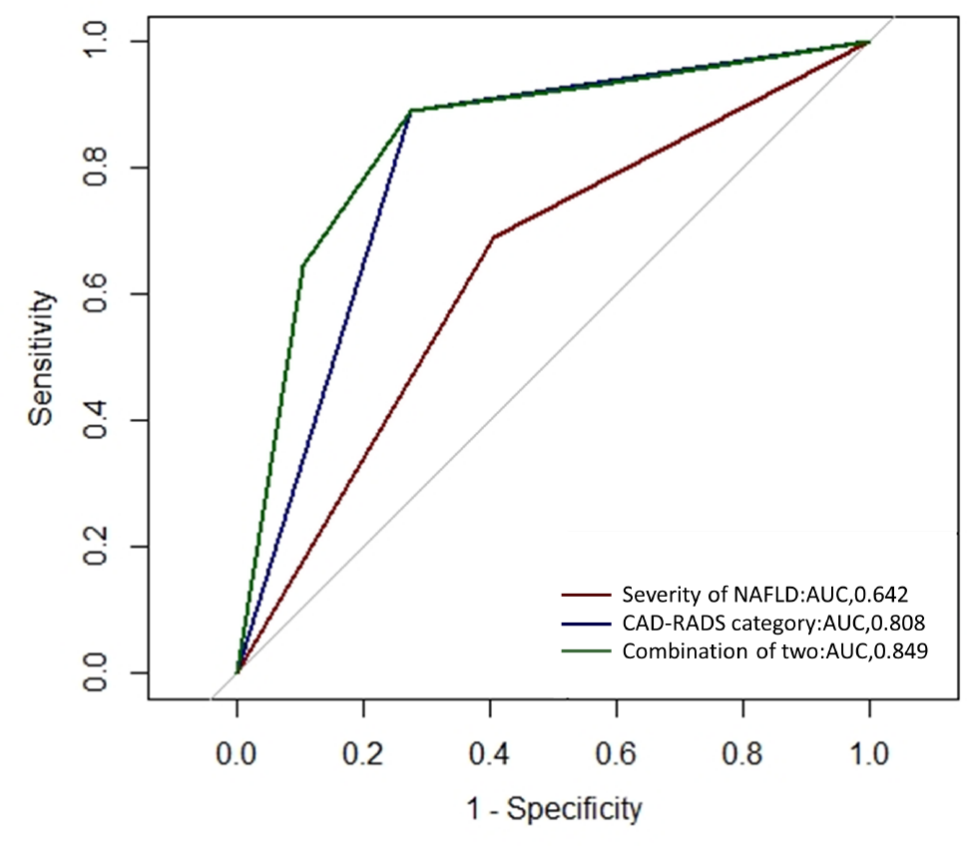
The predictive performance of severity of NAFLD and CAD-RADS category for the risk of MACEs.

The ROC curves of the severity of NAFLD and CAD-RADS category for predicting the risk of MACEs were shown in Fig. 3. The AUCs of the severity of NAFLD and CAD-RADS categories were 0.642 (95% CI = 0.557–0.727) and 0.808 (95% CI = 0.745–0.870), respectively. The combined ROC curve showed a good predicting performance, with an AUC of 0.849 (95% CI = 0.786–0.911). The most appropriate cutoff values of the combined ROC curve and CAD-RADS category curve only in predicting the risk of MACEs were set at 0.121 using the Youden index. The sensitivity and specificity were 88.9% and 72.6%, respectively.

### The severity of NAFLD was an independent predictor for MACEs in patients with CAD-RADS categories 3–5

During the follow-up times, MACEs were observed in 11 of 61 mild NAFLD patients (18.03%) and 29 of 60 moderate to severe NAFLD patients (48.33%) in patients with CAD-RADS categories 3–5. On univariate analysis, age (HR = 0.96; 95% CI = 0.93–0.99; *p* = 0.021), hypertension (HR = 0.51; 95% CI = 0.27–0.96; *p* = 0.036), and the severity of NAFLD (HR = 3.19; 95% CI = 1.59–6.42; *p* = 0.001) were associated with MACEs in patients with CAD-RADS categories 3–5. On multivariate analysis, the severity of NAFLD (HR = 2.94; 95% CI = 1.45–5.94; *p* = 0.003) was an independent predictor for MACEs. On the contrary, all factors had no statistical difference for MACEs in patients with CAD-RADS categories 0–2, including the severity of NAFLD (HR = 1.48; 95%CI = 0.23–9.45; *p* = 0.681) (Table 4).

**Table 4:** Cox regression analysis of variables for the association between severity of NAFLD and MACEs stratified according to CAD-RADS categories

Variables	Patients with CAD-RADS Categories 0–2 (*n* = 220)				Patients with CAD-RADS Categories 3–5 (*n* = 121)			
	Univariate		Multivariate		Univariate		Multivariate	
	HR (95%CI)	P Value	HR	P Value	HR (95%CI)	P Value	HR	P Value
Age(y)	1.03(0.95–1.12)	0.437	NA		0.96(0.93–0.99)	0.021	0.98(0.95–1.01)	0.105
Men	2.93(0.49–17.60)	0.241	NA		1.37(0.74–2.57)	0.318	NA	
**Risk of factor**								
History of smoking	2.32(0.26–20.86)	0.453	NA		1.78(0.93–3.42)	0.082	NA	
Hypertension	40.50(0.01-212365.09)	0.397	NA		0.51(0.27–0.96)	0.036	1.61(0.84–3.08)	0.149
Diabetes mellitus	0.65(0.71–5.99)	0.707	NA		0.87(0.47–1.62)	0.658	NA	
History of coronary heart disease	3.61(0.60-21.73)	0.161	NA		0.61(0.29–1.28)	0.192	NA	
Medication history	0.94(0.10–9.07)	0.959	NA		1.22 (0.63,2.37)	0.563	NA	
Family history	0.04(0.00-56047.593)	0.660	NA		0.65(0.20–2.10)	0.467	NA	
**Laboratory tests**								
AST (U/L)	1.00(0.96–1.06)	0.675	NA		1.00(0.98–1.02)	0.813	NA	
ALT (U/L)	1.01(0.98–1.03)	0.905	NA		1.01(0.99–1.02)	0.495	NA	
GGT (U/L)	1.00(0.99–1.02)	0.754	NA		1.00(0.98–1.01)	0.497	NA	
TG (mmol/L)	0.85(0.36–2.04)	0.720	NA		1.01(0.82–1.24)	0.939	NA	
TC (mmol/L)	0.95(0.45–2.03)	0.900	NA		0.89(0.70–1.13)	0.352	NA	
HDL-C (mmol/L)	0.38(0.01–15.52)	0.611	NA		0.76(0.22–2.69)	0.672	NA	
LDL-C (mmol/L)	0.99(0.37–2.64)	0.984	NA		0.92(0.64–1.31)	0.640	NA	
GLU (mmol/L)	1.11(0.77–1.60)	0.586	NA		1.00(0.90–1.12)	0.968	NA	
BUN(mmol/L)	1.00(0.86–1.15)	0.970	NA		1.19(0.96–1.46)	0.114	NA	
CREA(µmol/L)	1.02(0.99–1.05)	0.153	NA		1.02(1.00-1.03)	0.124	NA	
URIC(µmol/L)	1.01(1.00-1.02)	0.225	NA		1.00(1.00-1.01)	0.151	NA	
**Severity of NAFLD**	1.48(0.23–9.45)	0.681	NA		3.19(1.59–6.42)	0.001	2.94(1.45–5.94)	0.003

*Abbreviations* HR, hazard ratio; CI, confidence interval; NAFLD, nonalcoholic fatty liver disease; MACEs, major adverse cardiovascular events; CAD-RADS, Coronary Artery Disease Reporting and Data System; ALT, alanine aminotransferase; AST, aspartate aminotransferase; GGT, gamma glutamyl transferase; TG, triglyceride; TC, total cholesterol; HDL-C, high-density lipoprotein cholesterol; LDL-C, low-density lipoprotein cholesterol; GLU, glucose; BUN, blood urea nitrogen; CREA, creatinine; URIC, uric acid

**Fig. 4:**
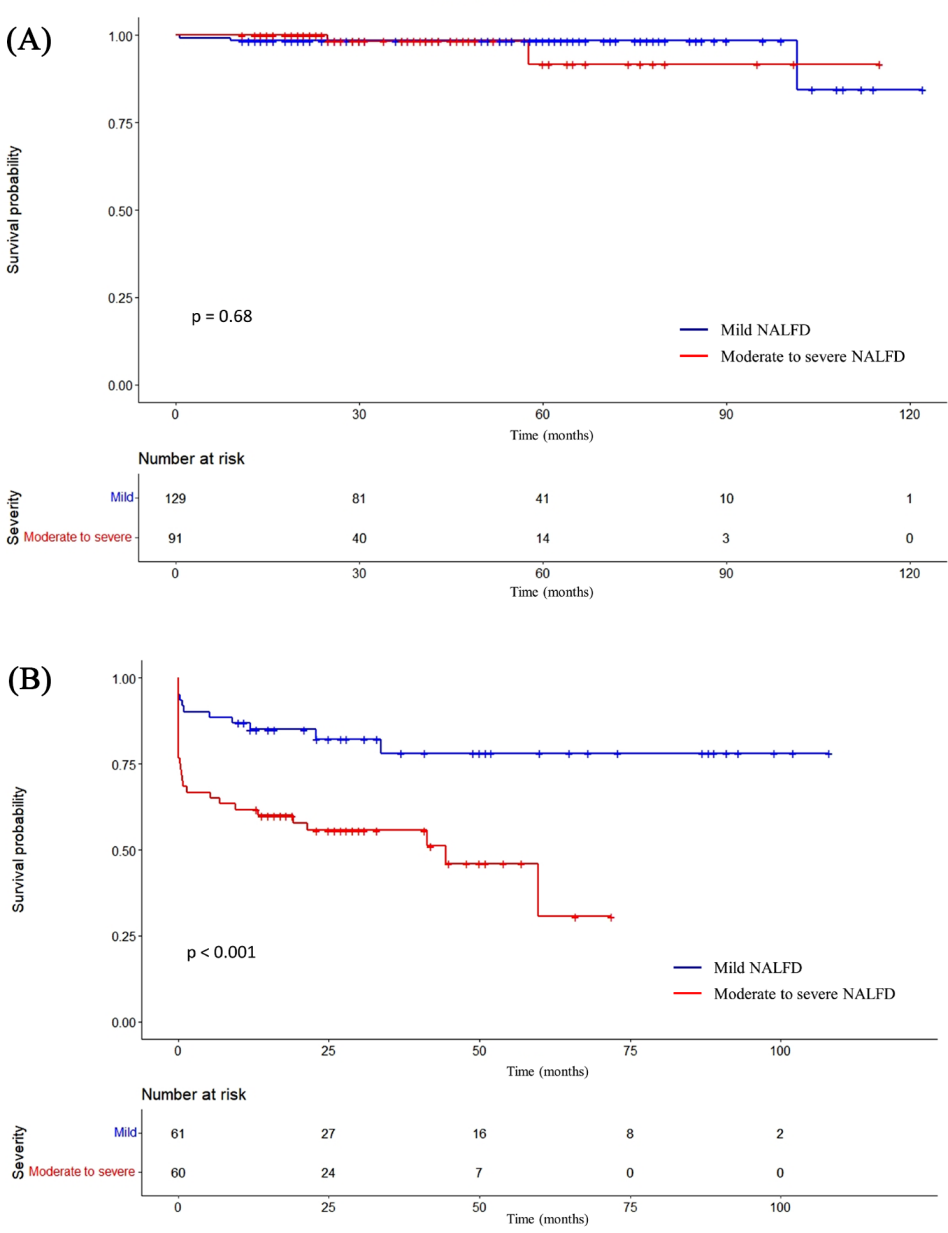
Kaplan–Meier curves of MACEs stratified according to CAD-RADS categories at CCTA in patients with mild NAFLD and moderate to severe NAFLD. (**A**) Survival rate of moderate to severe NAFLD patients compared with mild NAFLD patients in the CAD-RADS categories 0–2. (**B**) Survival rate of moderate to severe NAFLD patients compared with mild NAFLD patients in the CAD-RADS categories 3–5

The Kaplan–Meier curves analysis was used to show the survival rate. There was not statistical difference between the mild NAFLD patients and moderate to severe NAFLD patients with CAD-RADS categories 0–2 (log-rank *P* = 0.68) (Fig. 4A). The moderate to severe NAFLD patients had a poor prognosis compared with mild NAFLD patients with CAD-RADS categories 3–5 (log-rank *P* < 0.001) (Fig. 4B). The 1-, 3-, and 5-year survival rates of moderate to severe NAFLD patients were significantly lower than mild NAFLD patients with CAD-RADS categories 3–5 (61.7%, 55.7%, and 30.6% vs. 85.0%, 78.1%, and 78.1%, respectively).

## Discussion

In the present study, the severity of NAFLD was independent risk factor for MACEs in patients assessed by CCTA and moderate to severe NAFLD patients had a lower survival rate than mild NAFLD patients. The combined ROC curve of the severity of NAFLD and CAD-RADS category showed a good predicting performance for the risk of MACEs in patients assessed by CCTA.

Previous study reported that NAFLD was associated with calcified plaque, which is an independent predictor of CAD and cardiovascular outcome [17, 18]. Our results found moderate to severe NAFLD patients had lower calcified plaque volume and calcified plaque volume ratio than mild NAFLD patients. The reason might be that the aggravation of NAFLD severity may lead to liver dysfunction, insulin resistance, oxidative stress, arterial intimal dysfunction, and lipid metabolism abnormalities. When coronary artery is involved, forming atherosclerosis and coronary plaque, the number of plaques containing lipid components would also increase. Of course, the detailed association and mechanisms need to be further explored and confirmed through large sample and basic research. Previous study showed that the severity of NAFLD is associated with metabolic syndrome, including hypertension, liver aminotransferase, and dyslipidemia et al. [19]. Our results found that the ALT, AST, TC, and LDL-C levels were correlated with the severity of NAFLD according to the Spearman analysis. Though with limited roles in predicting the severity of NAFLD, increasing levels of these serum biomarkers may represent the mechanistic link with the severity of NAFLD [20], which further basic research is needed to confirm. FAI, as a part of the epicardial adipose tissue, could reflect the inflammation of the coronary artery on CCTA. Previous studies has been demonstrated that FAI is related to NAFLD, which our results was similar with it [21]. Epicardial adipose tissue also was associated with coronary artery disease assessed by CCTA. Previous studies demonstrated importance of epicardial adipose tissue volume to identify increased high-risk plaque and pericoronary adipose tissue inflammation [22]. However, the data of epicardial adipose tissue volume are not available in our hospital. It need to be studied further in the future.

It has been demonstrated that poor prognosis of liver-related disease increases progressively with the severity of NAFLD [23, 24]. A recent Swedish nationwide cohort study including 10,568 adults who were confirmed as NAFLD by liver histopathology suggested that the high all-cause mortality increased progressively with the severity of NAFLD [25]. However, much less is known about the association between the severity of NAFLD and poor cardiovascular outcomes. The major finding of the current study was that the severity of NAFLD was independent risk factor for MACEs in patients assessed by CCTA. Previous studies showed that the remission of severity of NAFLD may ultimately improve the prognosis of cerebrovascular and cardio-metabolic disease [6, 26]. It suggested that the severity of NAFLD plays an important role in poor cerebrovascular and cardiovascular outcomes, including the MACEs.

Our study clearly showed that the severity of NAFLD can provide important prognostic information for patients who underwent CCTA. Moderate to severe NAFLD patients had significantly lower survival rates than mild NAFLD patients during the follow-up period. We considered that the result mainly was related to the liver fat content of patients. Previous epidemiological studies reported that high liver fat content is likely to have a high risk of ischemic heart disease [27, 28] and impaired myocardial metabolism [5]. The potential mechanisms may be that severe NAFLD may contribute to MACEs by stimulating platelet activation or by aggravating systemic inflammation, liver insulin resistance, macrophage activation, increased oxidative stress, endothelial dysfunction, and altered lipid metabolism [29, 30]. Therefore, it is important to evaluate the severity of NAFLD, which may provide the opportunity to prevent progress of related complications and to reduce incidence of MACEs.

Moreover, we also evaluated predicting performance of the severity of NAFLD and CAD-RADS category for the risk of MACEs. The combined ROC curve of the severity of NAFLD and CAD-RADS category showed a good predicting performance with an AUC of 0.849, which was higher than the severity of NAFLD only and CAD-RADS category only, with AUC of 0.642 and 0.808, respectively. These results suggested that simultaneously evaluating the severity of NAFLD and CAD-RADS category would improve the detection of patients at higher risk of adverse cardiovascular events. Because these patients need more active medical strategies to alleviate symptoms and further improve prognosis [31].

It is vital to early identify and manage patients at high risk for different severity of NAFLD. The severity of NALFD was a significant independent predictor for MACEs in patients with CAD-RADS categories 3–5 in our study. Notably, our results showed that moderate to severe patients with CAD-RADS categories 3–5 have a relatively worsen prognosis and the survival rates reduce by about 70% within five years, which further elucidated the importance of assessing timely severity of NAFLD in patients with CAD-RADS categories 3–5 for reducing the risk of MACEs.

This study has several limitations. Firstly, this is a single-center retrospective study, and the small sample size, which might take inevitable selection bias. Secondly, the CT scanning images are obtained from different scanners, and lifestyle modification and medical therapies can modify severity of NAFLD. These issues need to be further settled in a future mulita-center prospective study with a large sample.

## Conclusion

In conclusion, this study showed that the severity of NAFLD was independent risk factor for MACEs in with obstructive CAD, having CAD-RADS 3–5 categories on CCTA. And moderate to severe NAFLD patients had a worsen prognosis than mild NAFLD patients. Early assessing the severity of NAFLD is important and it may help clinicians formulate individualized treatment.

### Electronic supplementary material

Below is the link to the electronic supplementary material. (no find link)

Supplementary Material

## Data Availability

The data sets generated and/or analyzed during the current study are available from the corresponding author on reasonable request.

## Abbreviations

ALT: Alanine aminotransferase
AST: Aspartate aminotransferase
AUC: Area under the curve
CAD: Coronary artery disease
CAD-RADS: Coronary Artery Disease Reporting and Data System
CCTA: Coronary computed tomography angiography
CI: Confidence interval
CREA: Creatinine
FAI: Fat attenuation index
GGT: Gamma glutamyl transferase
GLU: Glucose
HDL-C: High-density lipoprotein cholesterol
HR: Hazard ratio
ICC: Intraclass correlation coefficient
LDL-C: Low-density lipoprotein cholesterol
MACEs: Major adverse cardiovascular events
NAFLD: Nonalcoholic fatty liver disease
ROC: Receiver operating characteristic
TC: Total cholesterol
TG: Triglyceride
URIC: Uric acid
