# Supplementary material for: Association between severity of nonalcoholic fatty liver disease and major adverse cardiovascular events in patients assessed by coronary computed tomography angiography
Source: BMC Cardiovasc Disord. 2024 May 21;24:267. doi: 10.1186/s12872-024-03880-5 (PMC11107064; doi:10.1186/s12872-024-03880-5)
Supplement: Supplementary file 1 — Supplementary Material [file 12872_2024_3880_MOESM1_ESM.docx]

**E1 Coronary Computed Tomography Angiography Image Acquisition**

Routine coronary computed tomography angiography (CCTA) dates were obtained by dual-source CT scanner (Aquilion ONE, TOSHIBA; Somatom Flash or Force, Siemens Healthineers). Before scanning, all patients sprayed nitroglycerin sublingually to dilate the coronary artery and received breath-holding training to reduce respiratory motion artifacts. CCTA was performed by using a bolus tracking technique, with regions of interest placed in the root of the aorta. When the CT attenuation value reaches 100 HU, the scan would be automatically triggered after a delay of 5s. The non-ionic contrast agent and saline with 30-55 ml were injected into the peripheral vein on the back of the hand at a flow rate of 4-5 ml/s. CCTA images were collected using the following parameters: collimator=192 × 0.6 mm, layer thickness=0.75 mm, rotation time=0.25 s/circle, tube voltage 70~120 kV. The CT system automatically reconstructs the data of the best diastole and the best systole with the 0.75 mm layer thickness and 0.5mm section interval, and the convolution core was set to BV40.

**E2 Image evaluation details**

A dedicated plaque analysis software (Coronary Plaque Analysis, version 5.0.0, Siemens Healthineers, Germany) was used to measure the quantitative plaque parameters. The following parameters were recorded: (1) lesion length, from the proximal shoulder of plaque to the distal shoulder, was measured on curved reconstruction images in the best projection view; (2) total plaque volume; (3) calcified plaque volume; (4) calcified plaque volume ratio; (5) lipid plaque volume; (6) lipid plaque volume ratio; (7) fibrotic plaque volume and (8) fibrotic plaque volume ratio. The software automatically recognizes the centerline, outer contour, and inner contour of the lumen and automatically calculated the above-mentioned indicators.

The quantitative coronary perivascular fat attenuation index (FAI) was evaluated by using a dedicated FAI analysis software (Easy FAI, version 1.2, ShuKun, China).FAI was defined as the radial distance from the vessel wall equal to the vessel diameter for all voxels in the range of −190 to −30 HU. The software automatically calculates the attenuation values of perivascular adipose tissue at the proximal 40-mm segments of the left anterior descending artery and left circumflex artery and the proximal 10-50 mm segments of the right coronary artery. In all cases, it will be adjusted manually if needed.
